# Novel *in vitro* Experimental Approaches to Study Myelination and Remyelination in the Central Nervous System

**DOI:** 10.3389/fncel.2021.748849

**Published:** 2021-10-14

**Authors:** Davide Marangon, Nicolò Caporale, Marta Boccazzi, Maria P. Abbracchio, Giuseppe Testa, Davide Lecca

**Affiliations:** ^1^Laboratory of Molecular and Cellular Pharmacology of Purinergic Transmission, Department of Pharmaceutical Sciences, Università degli Studi di Milano, Milan, Italy; ^2^Department of Oncology and Hemato-Oncology, University of Milan, Milan, Italy; ^3^Human Technopole, Milan, Italy; ^4^Laboratory of Molecular and Cellular Pharmacology of Purinergic Transmission, Department of Pharmacological and Biomolecular Sciences, Università degli Studi di Milano, Milan, Italy

**Keywords:** myelination, oligodendrocytes, co-cultures, hiPSCs, organoids, models, neurodegenerative diseases

## Abstract

Myelin is the lipidic insulating structure enwrapping axons and allowing fast saltatory nerve conduction. In the central nervous system, myelin sheath is the result of the complex packaging of multilamellar extensions of oligodendrocyte (OL) membranes. Before reaching myelinating capabilities, OLs undergo a very precise program of differentiation and maturation that starts from OL precursor cells (OPCs). In the last 20 years, the biology of OPCs and their behavior under pathological conditions have been studied through several experimental models. When co-cultured with neurons, OPCs undergo terminal maturation and produce myelin tracts around axons, allowing to investigate myelination in response to exogenous stimuli in a very simple *in vitro* system. On the other hand, *in vivo* models more closely reproducing some of the features of human pathophysiology enabled to assess the consequences of demyelination and the molecular mechanisms of remyelination, and they are often used to validate the effect of pharmacological agents. However, they are very complex, and not suitable for large scale drug discovery screening. Recent advances in cell reprogramming, biophysics and bioengineering have allowed impressive improvements in the methodological approaches to study brain physiology and myelination. Rat and mouse OPCs can be replaced by human OPCs obtained by induced pluripotent stem cells (iPSCs) derived from healthy or diseased individuals, thus offering unprecedented possibilities for personalized disease modeling and treatment. OPCs and neural cells can be also artificially assembled, using 3D-printed culture chambers and biomaterial scaffolds, which allow modeling cell-to-cell interactions in a highly controlled manner. Interestingly, scaffold stiffness can be adopted to reproduce the mechanosensory properties assumed by tissues in physiological or pathological conditions. Moreover, the recent development of iPSC-derived 3D brain cultures, called organoids, has made it possible to study key aspects of embryonic brain development, such as neuronal differentiation, maturation and network formation in temporal dynamics that are inaccessible to traditional *in vitro* cultures. Despite the huge potential of organoids, their application to myelination studies is still in its infancy. In this review, we shall summarize the novel most relevant experimental approaches and their implications for the identification of remyelinating agents for human diseases such as multiple sclerosis.

## Introduction

Myelin is a very specialized lipidic insulating structure that allows rapid and efficient conduction of nerve impulses. During evolution, myelin has become a key requirement for the motor, sensory and cognitive functions in vertebrates (Zalc, 2006). Myelin sheath is the result of the complex packaging of multilamellar extensions generated by specialized glial cells, i.e., oligodendrocytes (OLs) in the central nervous system (CNS) and Schwann cells in the peripheral nervous system. Although anatomical and histological studies have described the existence of what now we call myelin since the 18th century, the structural and chemical complexity of myelin made the elucidation of its function elusive until recently (Boullerne, 2016).

Advances in understanding the physiology of myelination have been achieved only a few decades ago, when dissociated brain tissues from small mammals were cultured giving rise to the first primitive neuron-glia co-cultures (Bornstein and Murray, 1958; Kim, 1972). These studies paved the way to the modern myelinating *in vitro* models described in this review. In the CNS, the identification of OL precursor cells (OPCs) was the starting point for investigating oligodendroglia biology. OPCs, highly dividing cells during embryogenesis, become relatively quiescent (but not silent) during adulthood, when they still represent an important source of potential remyelinating OLs after injury (Nishiyama et al., 2021a). During their differentiation to mature myelinating cells, OPCs make contacts with other glial cells and neurons with which they can form synapses and gap junctions, and they continuously refine their intrinsic program based on extracellular cues and inputs received from contacted cells. Neurotransmitters, axonal signals, morphogens, cytokines, and extracellular matrix (ECM) proteins can all contribute to changing the fate of OPCs, modulating their maturation timing, promoting their local migration, or keeping them undifferentiated to maintain a pool of slowly proliferating cells (Nishiyama et al., 2021a). The cellular and molecular context make OPCs highly heterogeneous and plastic, both in time and space, with mechanisms that remain largely unknown. The recent development of 3D scaffolds and new materials with different stiffness has highlighted that, as any other cells, OPCs and OLs are also sensitive to the mechanical features of the extracellular environment. Thus, when grown on plastics, *in vitro* cultures may lead to more artifactual results than previously expected. For these reasons, isolation methods and culture conditions are a crucial issue to determine OPC behavior *in vitro*.

Although OPC monocultures recapitulate several features of OL differentiation and myelination, thus helping to elucidate myelin-related functions (Zuchero et al., 2015), they do not represent a proper myelination model, since in the absence of axons or axon-like structures they show limited myelination abilities. Rodent OPC-neuron co-cultures, still representing the most widely used myelinating assays, have intrinsic limits due to the potential species-specific differences in both differentiation and myelination dynamics (Mabbott et al., 2006; Scantlebury et al., 2014). Indeed, although myelin plays the same role in humans and rodents, myelination kinetics are significantly different. In rodents, myelination takes place after birth and is resolved within postnatal day 60, whereas in humans it requires more than 20 years (Nakagawa et al., 1998; Giedd et al., 1999; Yeung et al., 2014). This very long time is likely due to the unique complexity of human connections. Intrinsic differences in oligodendroglia biology between humans and rodents is also reflected in differences in the OPC pool (Dietz et al., 2016). For this reason, cellular reprogramming from human derived cells (typically dermal fibroblasts) into induced pluripotent stem cells (iPSCs) and then into terminally differentiated OLs is increasingly becoming an important means to assess the effect of pharmacological agents on OPC maturation and axonal myelination both in physiological conditions and after insults. Induced humanized models are likely to mimic more accurately the behavior of human cells. iPSCs can be derived directly from patients to assess the importance of genetic and/or environmental cues in the pathogenesis of diseases, and offer the opportunity to generate personalized disease models. A few examples have been described also in demyelinating diseases such as leukodystrophies or multiple sclerosis (MS). Human iPSCs can be also grown as organoids, self-organizing 3D structures that recapitulate the developmental features of the organ of origin. Brain organoids have been used to study the formation of cortical layers maintaining the complex cell-to-cell interactions, with a strong focus on neurons, whereas OL and myelination dynamics have been mostly neglected.

**FIGURE 1:**
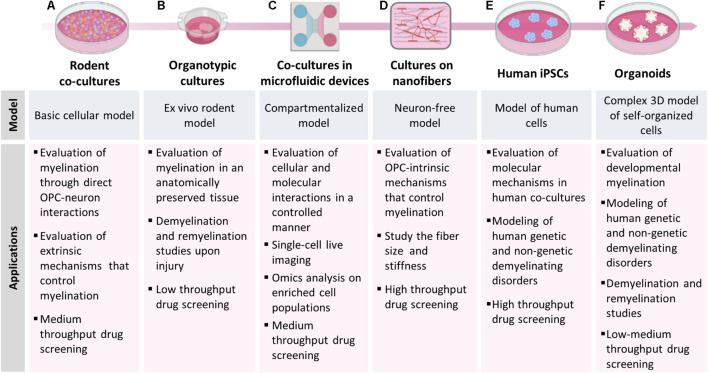
Models to study myelination, demyelination and remyelination. The picture shows the different models of myelination described in the manuscript and their main applications. **(A)** Rodent co-cultures are easy to set-up, relatively inexpensive and can be used to study mechanisms involving OPC-neuron interactions and to evaluate the effect of candidate compounds in low-medium throughput drug screening. **(B)** Organotypic cultures allow to perform myelination studies in a more complex and anatomically preserved environment; the slices can be isolated from transgenic mice for fate-mapping studies, demyelinated with toxicants and used to test the remyelinating potential of candidate compounds. **(C)** Microfluidic devices induce neurons to project their axons into an adjacent compartment and form an aligned dense neurite network, on which OPCs can maturate and produce myelin; this model is particularly useful for single-cell live imaging analysis. **(D)** OPCs can also be cultured on bio-compatible nanofibers to study OPC-intrinsic mechanisms that control myelination. **(E)** The use of iPSCs from human subject allows to establish human co-cultures made of iPSC-derived OLs and neurons or **(F)** to generate brain organoids, providing *in vitro* models to study human diseases.

In this review, we shall describe the classic methods used to study myelination and remyelination *in vitro*, we shall introduce some of the most recent methods, their limits, and their potential relevance in the field (Figure 1), and finally shall describe the most relevant implications in the identification of remyelinating agents for human diseases.

## Classic *In Vitro* Methods and Myelination Assays

The first reports showing *in vitro* myelination date back to the late 1950s and made use of various CNS tissues from chicks, kittens or rodents broken up by chemical and mechanical digestion and then dissociated to obtain a mixed cell population (Bornstein and Murray, 1958; Kim, 1972). The derived cultures were plated on collagen and cultured in high serum media giving rise to irregular myelination and to very variable experimental regimes. Subsequent improvements in culturing protocols of dissociated cells (e.g., development of defined media and usage of more compliant substrates) allowed to reduce variability and to greatly increase the control over experimental conditions. Moreover, technical advances allowed to define some fundamental aspects of myelination dynamics, such as the ability of OL processes to exclusively myelinate axons, the absolute need of neurons and of a continuous bidirectional crosstalk between neurons and OLs to allow a proper myelination, the synchronization of these processes, the importance of axonal density, the role of additional cells like astrocytes or microglia (Raffaele et al., 2021b) and the similarities and differences between *in vitro* and *in vivo* myelination (Lubetzki et al., 1993; Jarjour et al., 2012). Dissociated CNS cultures from different brain areas (e.g., hippocampus, spinal cord, cerebellum) allowed to identify, for the first time, region-specific differences in myelination dynamics (Baumann and Pham-Dinh, 2001; Thomson et al., 2006). Although dissociated CNS cultures are made by both neurons and glial cells, their common source does not allow to independently manipulate the different populations. Furthermore, the highly inconsistent neuron survival from study to study may lead to highly variable results.

Myelinating co-culture models in which neurons and OLs are independently isolated and then cultured together have been developed to overcome some of the limitations described above (Figure 1A). In these models, neurons are usually isolated from embryonic brain tissues, and then differentiated to reach morphological and functional maturation before adding oligodendroglial cells. On the contrary, OPCs are usually isolated after birth, before the end of the first postnatal week, when they reach the highest density (Nishiyama et al., 2021b), so that they can be expanded using mitogens (e.g., bFGF and PDGF) and then plated onto mature neurons. OLs and neurons can be obtained from different species, CNS regions, and tissues at different developmental stage, or they can be prepared using different methods (e.g., isolation from tissue explants or mixed glial cultures, immunopanning, magnetic or fluorescent-activated cell sorting). Moreover, both populations can be manipulated independently before setting up the co-culture. The co-culture of dissociated dorsal root ganglia neurons (DRGs) with primary oligodendroglial cells is one of the most used myelinating model in the last decades (Windebank et al., 1985; Xiao et al., 2009). In this model, DRGs from rodent embryos are removed from spinal nerves and maintained in culture until maturation (usually 3 weeks) and then OPCs from postnatal day 1–3 pups are added onto them. Recent refinements of the experimental protocols allowed to obtain myelinating fibers in 10–18 days after adding OLs to neurons (Azevedo et al., 2018; Shimizu et al., 2018). Even though OPCs/OLs are exclusively CNS glia while DRGs are not CNS neurons, this mixed co-culture contributed to identify both secreted (e.g., neuregulins, ATP) and contact-mediated (e.g., integrins) axonal signals influencing oligodendroglial development and myelination. However, analogies between *in vivo* CNS myelination and findings obtained with this model may be questionable. Moreover, DRG survival depends on the presence of neurotrophins (e.g., nerve growth factor), which can negatively affect axon myelination (Chan et al., 2004). To address these major issues several co-culture models were developed taking advantage of different types of neurons (hippocampal, cortical, cerebellar) isolated from CNS tissues of rodent embryos.

A method to obtain hippocampal neuron/OL co-cultures in the absence of an astrocyte feeding layer was described for the first time in 2012 (Gardner et al., 2012). These co-cultures were used to study myelin-mediated regulation of ion channel trafficking (Gardner et al., 2012), the mechanism defining myelination-incompetent axon segments during neural development (Diez-Revuelta et al., 2017) and to develop assays for the evaluation of drugs promoting CNS axon regeneration (Gang et al., 2015). Hippocampus contains many myelinated axons projecting within the hippocampus (Arnold and Trojanowski, 1996), whose alterations may profoundly affect learning and memory. Hippocampal demyelination is a prominent feature of MS brain, and hippocampal atrophy correlates with cognitive decline in MS patients (Baltan et al., 2021). Nevertheless, it has been shown that CA1 neurons may survive demyelination and hibernate in a state that protects the demyelinated axon, facilitating functional recovery following remyelination. Hippocampal demyelination was also observed in Alzheimer’s disease (Ota et al., 2019) and epilepsy (Drenthen et al., 2020), suggesting a contribution to the onset of several cognitive symptoms.

Analysis on MS post-mortem tissues highlighted signs of demyelination in many brain regions, including cortical and sub-cortical areas. Interestingly, in some cases, cortical demyelinated lesions did not show signs of immune cell infiltration, suggesting that neurodegenerative events may precede neuroinflammation and white matter demyelination (Trapp and Nave, 2008). In this context, cortical neuron/OL co-cultures may represent a useful system for studying the axo-myelinic neurotransmission and the evolution of MS lesions at cortical level. To date, this co-culture system has been used to investigate the myelin membrane trafficking pathways required for axon ensheathment (Feldmann et al., 2011) and the effect of OL-derived exosomes on neuronal firing rate, axonal transport, and signal transduction (Frohlich et al., 2014; Fruhbeis et al., 2020).

The co-culture models described above do represent a valuable tool for studying the fine mechanisms that regulate axon-OL interplay and myelination, but they do not consider either the three-dimensional structure of the CNS or the role of other CNS populations, which may affect the dynamics of myelination, demyelination and remyelination. On the contrary, animal models currently represent the most suitable available non-human models, but have limitations in terms of management, costs, feasibility of drug treatments and biotechnological manipulation. The golden mean between *in vitro* and *in vivo* models of myelination are *ex vivo* cultures, in which the three-dimensional structure of the different brain areas is reproduced taking advantage of thin slices (Figure 1B). The advantage of this system is that it can be easily used as disease model, since brain slices can be isolated from different regions of animal models of disease such as transgenic mice, (Humpel, 2019) and they can be demyelinated *in vitro* exposing the cultures to toxicants. For example, lysolecithin-treated slices have been widely used to study early demyelination events, response of OPCs to demyelinating insults, remyelination and the role of other glial populations, and to evaluate the remyelinating potential of pharmacological and biotechnological interventions (Miron et al., 2010; Goudarzi et al., 2016; Marangon et al., 2020).

## New Materials and Supports for Cell Cultures

The *in vitro* myelination models described above are made up by intricate networks of co-cultured cells, making it difficult to perform single-cell and large scales (omics) analyses on either neurons or myelinating cells. Microfluidic technology can be used to overcome these limitations by assembling multi-compartment cell culture devices in which microenvironmental conditions can be controlled spatially and temporally to better mimic *in vivo* conditions (Figure 1C). These devices allow the compartmentalization of neuronal cell bodies, axons and myelinating cells, and the generation of aligned dense networks in specific compartments that are physically isolated from neuronal somas, thus enabling single-cell live imaging, high-throughput and -omics analyses on highly enriched cell populations.

The first application of this technology to neuroscience dates back to 2005, when Taylor et al. (2005) isolated and directed the growth of CNS axons without neurotrophins by means of a microfluidic platform, providing a highly adaptable system to model many aspects of CNS physiopathology (Taylor et al., 2005). The culture chamber consisted of a polydimethylsiloxane (PDMS) mold containing a relief pattern of somal and axonal compartments (1.5-mm wide, 7-mm long, 100-mm high) connected by microgrooves (10-mm wide, 3-mm high), which guide axon growth into the axonal side. This method allowed to investigate the transport of axonal mRNAs in developing neurons, to test the role of soluble factors in axonal injury and regeneration, and to generate the first prototype of compartmentalized co-culture with oligodendroglial cells. However, only several years later this technology was successfully applied to study myelination, demyelination and remyelination.

For example, Vaquié et al. (2019) generated a lesion model of myelination suitable for high-resolution time-lapse imaging of neuron-glial cell interactions and transcriptomic analyses both in physiological conditions and after lesion (Vaquié et al., 2019). In this device, made of two chambers connected by microgrooves, dissociated DRGs were plated in the first chamber, from which neuronal cell bodies projected their axons to the second chamber where precursors of myelinating cells were plated. Authors showed that both OLs and Schwann cells were able to myelinate axons and that laser-induced axonal lesion in the microgrooves led to demyelination in the second chamber. Interestingly, in response to VEGFR1 activation, Schwann cells were able to guide axonal regrowth, re-interact with regenerated axons and remyelinate them, whereas OLs, which did not upregulate VEGFR1 after axonal lesion, passively responded to demyelination either dying or remaining in their original myelinating state (Vaquié et al., 2019). The regenerative action of Schwann cells depends on the formation of “actin spheres,” which constrict axons until their disintegration, thereby accelerating the clearing of axonal debris after lesion. Of note, enforced expression of VEGFR1 in OLs induced axon fragmentation to a similar level as in neuron/SC cultures and promoted axonal regrowth.

The advantages of microfluidic devices can be also combined with those offered by embryonic stem cells (ESCs) and iPSCs (see section “Models to Study Human Oligodendrocytes in Physiology and Disease”). In this context, in 2015, Kerman et al. (2015) developed a custom semi-automated computer platform, called Computer-assisted Evaluation of Myelin formation, to quantify myelin in co-cultures of ESC-derived myelinating OLs and cortical neurons plated on compartmentalized microfluidics-based devices (Kerman et al., 2015). Unlike the original microfluidic device design by Taylor, which is characterized by low-height cell compartments enclosed within PDMS, authors adopted an open-well design, which, by reducing the capillary forces, allowed to increase the number of neurons remaining in the neuronal compartment and the density of axons growing into the myelination compartment. In this study, ESCs were firstly converted into neural precursor cells (NPCs) (Marchetto et al., 2008) and then differentiated into OLs by switching them firstly in OPC differentiation medium (N2/B27 supplemented with IGF1 and PDGFα) and then in myelination medium (N2/B27 with CNTF, NT3 and T3), whereas neurons were differentiated from ESCs following a previously published protocol that enriched the cultures in cortical neurons (Gaspard et al., 2009). At 10–14 DIV, when neurons extended many axons into the myelination compartment, mature OLs were added to the device. Maintaining co-cultures in 40 ng/ml T3 significantly promoted myelination of axons. This assay allowed to follow the myelination process by real time imaging over several days and to clearly show that OLs sense the environment constantly extending and retracting their processes around and along axons before starting myelination.

Very recently, another group developed a new 3D printing-based fabrication method to produce repeatably PDMS-based microfluidics devices with an open compartment design (Ristola et al., 2019). These microfluidics devices were made by three sequential compartments: two neuronal soma compartments interconnected to a central compartment by forty 250 μm-long microtunnels. This characteristic architecture allowed the growth of an aligned neurite network in which neurite-OL interactions and myelination could be easily analyzed. To obtain a myelinating culture, primary DRG neurons isolated from spinal cords of E15 rat embryos were first seeded in the neuronal soma compartments and, after 21 DIV, OPCs were seeded onto the neurite network compartment of the device. After 18 days in co-culture in SATO medium, OLs showed MBP-positive segments along the neurites in an aligned configuration. Interestingly, the size of microtunnels and myelination compartments have been reported to influence both neurite outgrowth and myelination; indeed, neurons extended their neurites into the myelination compartment more efficiently in larger microtunnels (3.5-μm high and 10-μm wide) compared to smaller ones (1.5-μm high and 5-μm wide). Instead, a shorter myelination compartment (3-mm long) promoted the formation of more aligned and dense neurite networks with respect to larger one (5-mm long), facilitating myelination analysis.

Myelin wrapping is also closely related to axonal diameter. Large-diameter axons are preferentially myelinated over small-diameter ones, which remain unmyelinated (Voyvodic, 1989). However, understanding the exact contribution of axonal diameter to myelin formation by using co-culture systems remains complex, since the diameter-dependent mechanisms are barely distinguishable from those induced by the extrinsic cues deriving from axon-glia contacts. Several studies demonstrated that axonal signals mainly act as negative regulators of myelination, whereas the initiation of the process does not depend on axonal signals (Podbielska et al., 2013). Accordingly, OPCs seeded onto fixed axons differentiate and form compact myelin with the same timing and robustness of OPCs on live axons, demonstrating that dynamic interactions between OLs and axons are not required for either differentiation or myelination (Rosenberg et al., 2008). The same results were obtained several years later by using polystyrene nanofibers, inert structures designed to mimic CNS axons (Figure 1D). This important finding opened new horizons in exploring the autonomous OL cellular mechanisms that control myelination. In 2013, Lee et al. (2013) fabricated aligned fibers by electrospinning liquid polystyrene, a material commonly used for cell cultures, demonstrating that fibers with a >0.4 μm diameter represent a sufficient axonal cue for initiating myelin wrapping by OLs (Lee et al., 2013). Later, Bechler et al. (2015), taking advantage of parallel-aligned electrospun fibers composed of poly-L-lactic acid, showed that OLs do have the intrinsic capability to form compact sheaths that reflect their *in vivo* origin, demonstrating that OPCs acquire a regional identity prior to differentiation and that this identity determines sheath length (Bechler et al., 2015).

Thanks to advances in aligned electrospinning technology, nanofibers can now be rapidly fabricated, standardized, and configured into various densities and patterns as desired. OPC-nanofibers cultures can also be used to assess whether and how individual neuronal surface signals may modulate contact formation with OLs and their myelinating capacity. To this aim nanofibers are coated with signaling proteins of interest. OPCs plated on ephrin-A1-coated fibers exhibited a decreased maturation rate and generated fewer sheath-like structures per cell compared with poly-D-lysine-coated control fibers, suggesting that OL ephrin receptors are involved in regulating myelination by sensing inhibitory cues in the form of ephrins expressed on the axonal surface (Harboe et al., 2018).

A great advantage of the nanofiber system is that it facilitates the assessment of multiple biologically relevant parameters, providing key insight into which cellular process may be affected by any given treatment. Nevertheless, most existing high-throughput systems prioritize the speed of analysis and allow only to acquire information on single parameters, such as MBP intensity, sheath length, g-ratio. In this context, automated high-throughput methods that combine micro-engineered nanofibers, automated microscopy, and analytic algorithms to extract detailed morphological properties from individual OLs can be exploited to remove bias and variability, facilitate massive increases in sample size and analytic speed and to accelerate the discovery of new therapeutics with pro-myelinating activity (Xu et al., 2019).

The advancement in electrospinning technology and production of new biodegradable and biocompatible materials may also have unprecedented applications in neural regeneration. In this context, polycaprolactone (PCL), and PCL-gelatin copolymer nanofibers, represent interesting materials. Both the nanofibers supported OPC growth, survival, and differentiation, but differentiated OLs formed significantly more myelinated nanofiber segments with PCL-gelatin than PCL-only nanofibers, demonstrating that fiber composition, biological function and hydrophilicity can affect the efficiency of OPC differentiation and fiber wrapping (Li et al., 2014).

Chemical, physical and mechanical properties of the macroenvironment strongly influence basic processes such as proliferation, maturation, migration, response to stimuli in a similar way to biochemical signals (Jagielska et al., 2012). This issue has been largely overlooked until recently. Brain tissues and axons have an extremely low mechanical stiffness (Young’s elastic modulus E ∼ 0.1–1 kPa), which is approximately six orders of magnitude lower than silica glass, polystyrene, and PCL (Young’s elastic modulus 10^8–9^ Pa). Interestingly, stiffness of some regions of the brain have a significant linear correlation with age and sex, while others do not (Arani et al., 2015; Takamura et al., 2020). Espinosa-Hoyos et al. (2018) developed the first mechanically compliant axon-like fiber arrays, a model that replicates the physical cues of the glial cell microenvironment (Espinosa-Hoyos et al., 2018) and that allows to evaluate the effects of axon diameter, artificial axon stiffness and ligand coating on OL engagement and myelination. They have demonstrated that OLs were three times more likely to ensheath stiffer (140 kPa) artificial axons, compared to more compliant counterparts approximating the sub-kPa stiffness of biological axons (0.4 kPa). Furthermore, in line with previous findings, they found that laminin-coated artificial axons were more significantly ensheathed by OLs compared to poly-D-lysine-coated artificial axons and that OLs preferentially engaged 10-μm diameter axons compared to 20-μm ones, suggesting a maximum permissive diameter threshold above which full myelin wrapping may not proceed efficiently. This study revealed that both axon diameter and ligand type can play a role in OL response in the presence of axon-like mechanical stiffness and it may represent the basis for the generation of engineered environments that reflect key pathophysiological, mechanical, geometric, and biochemical components of the glial microenvironment for myelination assays.

The efficiency of OL differentiation and myelination depends on ECM stiffness as well. Indeed, a more rigid ECM results in significant reduction in OL branching complexity, which strongly correlates with decreased expression of OPC differentiation markers (Urbanski et al., 2016). Thus, changes in the mechanical properties of the CNS following injury may play a prominent role in repair and remyelination. It has been recently shown that acutely demyelinated lesions, which can spontaneously remyelinate, present lower stiffness than healthy tissue in both the lysolecithin and cuprizone models, whereas tissues chronically exposed to cuprizone are stiffer than healthy white matter (Urbanski et al., 2019), suggesting that the increase of matrix rigidity observed in chronic MS lesions might be involved in remyelination failure. The effect of ECM stiffness on oligodendroglial functions has been recently studied during aging. OPC microenvironment stiffens with age and this mechanical change is sufficient to cause age-related loss of function of OPCs (Segel et al., 2019). Aged OPCs cultured on synthetic scaffolds, designed to mimic the stiffness of young brains, or transplanted into the prefrontal cortex of neonatal rats, were molecularly and functionally rejuvenated, whereas young progenitors transplanted into aged environments lost optimal regenerative properties, indicating that ECM aging contributes to the impairment of OPC functions. Interestingly, this age-related loss of function can be reversed also through inhibition of PIEZO1 (Segel et al., 2019), a mechanosensitive ion channel which regulates cell density and stem-cell activation. Piezo1 knockdown in aged mice after lysolecithin-induced demyelination markedly improved the regenerative capacity of OPCs, whereas its knockdown in pups increased the total density of OPCs, suggesting that PIEZO1-mediated mechanical signaling is essential for OPCs during growth and development, but then becomes non-compliant to regeneration with aging.

## Unraveling Oligodendroglial Heterogeneity With Single-Cell Omics

The recent development of single-cell omics represents a unique opportunity to quantify simultaneously a high number of molecular states for each cell of a complex organ. It is now possible to genome-wide profile, at different degrees of sensitivity, accuracy and scalability, RNA, DNA, histone modifications, chromatin accessibility, DNA methylation, nuclear lamina interactions, chromosomal contacts, and the protein signatures of individual cells. Among these, single cell RNA seq is at the forefront because of the higher throughput and accuracy, and its application to the clinics is now closer (Tanay and Regev, 2017; Chappell et al., 2018). These advances have opened the possibility to overcome the inherent limitations associated with the averaged readout of bulk sequencing for mixtures of heterogeneous cell populations and thereby unveil how cell-types expressing the same canonical markers may instead comprise more diverse subpopulations than previously thought.

For a long time, OPCs have been considered as a functionally homogeneous population in the adult CNS. However, as highlighted above, when looking at the whole oligodendroglial lineage, they show significant differences in terms of proliferation, differentiation, myelination, migration capabilities and energetic metabolism. Moreover, a large fraction of OPCs does not differentiate, but instead remains in a progenitor state throughout adulthood, suggesting the existence of sub-populations with different functions. OPCs from different brain regions express distinct ion channel profiles, which lend unique electrical properties (Spitzer et al., 2019). Conditional loss of OPCs in specific brain regions led to unexpected outcomes, such as depressive-like behavior, persistent weight-gain, and leptin insensitivity. Moreover, in specific conditions, OPCs can also display immunomodulatory properties, upregulating the antigen presentation machinery and cytokine production (Fernandez-Castaneda et al., 2020). The dynamic role of OPCs in the adult CNS was recently investigated by exploring adult OPC diversity in the mouse brain at transcriptional level (Beiter et al., 2020). OPCs from the adult mouse brain clustered into three distinct subpopulations characterized by specific transcriptional signatures and Gene Ontology profiles. Interestingly, OPC cluster 1 (OPC1) and 3 (OPC3) expressed a unique subset of common OPC markers, suggesting that they may not be critical to OPC functioning as previously thought. OPC1 expressed high levels of Clusterin, a gene found upregulated in both Alzheimer’s disease and MS, suggesting that these cells may play an active role in these diseases. OPC cluster 2 showed enrichment in multiple proteins involved in ECM organization and cytokine mediated signaling, whereas OPC3 showed significant upregulation of the G protein-coupled receptor (GPR17), a promising target for remyelination therapies (Bonfanti et al., 2020; Parravicini et al., 2020; Angelini et al., 2021) and up-regulation of several genes related to neuronal differentiation and synapse organization.

Mature OLs are heterogeneous as well. This was recently highlighted analyzing more than 5.000 individual cell transcriptomes expressing markers from the OL lineage, isolated from 10 distinct regions of the anterior-posterior and dorsal-ventral axis of the mouse juvenile and adult CNS (Marques et al., 2016). Biclustering analysis, hierarchical clustering, and differential expression analysis led to the identification of six distinct populations of mature OLs (MOL) and two populations of myelin-forming OLs. However, despite some mature OL populations were found enriched in certain regions in juvenile mice, they disappeared in adulthood, suggesting that each brain region optimizes its circuitries by a unique proportion and combination of mature OLs during development. Differentially and equally expressed genes among these populations indicated segregation of MOL1-4, enriched in lipid biosynthesis and myelination genes, from MOL5-6 clusters, enriched for synapse components.

In addition to advancing our understanding of the molecular mechanisms underlying OL functions, single cell omics studies represent a powerful tool for studying the pathogenesis of OL disorders. Single cell transcriptomic analysis of the OL lineage in mice recently allowed to pinpoint an active role of OLs in the paradigmatic case of MS. On one hand, Falcao et al. (2018) discovered that during disease, OLs express genes of the major histocompatibility complex class I and II (MHC-I and –II), and a substantial number of susceptibility genes for MS, so far mainly associated with immune cells. Indeed, they next showed that OPCs can phagocytose and activate memory and effector CD4-positive T cells (Falcao et al., 2018). On the other hand, Moyon et al. (2015) found that adult OPCs revert to a neonatal-like transcriptome when activated during MS, by increasing innate immune system genes, IL1β and CCL2, and producing cytokines that increase their capacity to migrate to areas of demyelination and thus have an impact on the process of remyelination (Moyon et al., 2015).

One of the most interesting potentials of single-cell omics data analysis is the reconstruction and analysis of developmental trajectories. Single cell transcriptomes can be projected, after dimensionality reduction, in a common analytical space through different algorithms that can define a distance between the single cells coming from a complex tissue based on the similarity of their transcriptomes, thus defining a new temporal concept defined as pseudotime (Haghverdi et al., 2016; Tritschler et al., 2019). This is recently acquiring even a dynamic spin, with the introduction of the concept of RNA velocity, which models the derivative of mRNA abundance through an estimate of spliced vs. unspliced transcripts, and thus infers the direction of transcriptional regulation between cells in the pseudotime trajectory (La Manno et al., 2018; Gorin et al., 2020). Jakel et al. (2019) took advantage of snRNA-seq and Single-cell Near-Neighbor Network Embedding analysis, which places transcriptionally similar clusters close to each other on a tree, to identify new oligodendroglial populations in pseudotime in the human white matter of control subjects and progressive MS patients. Thanks to this approach, OL lineage cells were segregated into 7 oligodendroglial (Oligo 1–6 and imOL, immune oligodendroglia), one OPC and one committed OL precursor clusters (Jakel et al., 2019). Pseudotime analysis identified Oligo6 as an intermediate stage between OPCs and mature OLs, and Oligo1 and Oligo5 as end-states. Interestingly, the transcriptional network of the latter relates cell-cell adhesion and viability, whereas they did not present high expression of myelin genes (as Oligo3 and Oligo4 cluster), suggesting that not all mature OLs maintain a strongly active transcriptional machinery for myelination. The intermediate Oligo6 cells, which are extremely enriched in Opalin, and Oligo1, were found significantly reduced in MS, both in normal-appearing white matter and lesions, adding evidence to the concept that normal-appearing white matter is indeed not “normal” (de Groot et al., 2013; Angelini et al., 2021). On the contrary, Oligo2, Oligo3, Oligo5 and imOLs, which are closely associated with microglia, have been found enriched in MS, suggesting that MS pathophysiology may not be strictly associated with a global failure of differentiation, but rather with a skew in specific subpopulations.

Specifically, single cell analysis has also enabled fundamental advances of our understanding of human OL development, suggesting a higher cellular heterogeneity than previously thought, with defined subpopulations and cell states associated with different stages of lineage progression and functional states (van Bruggen et al., 2017).

As a matter of fact, single-cell transcriptome profiling of cortical lobes and pons during human embryonic and fetal development allowed to capture the developmental trajectory of OL lineage cells until the human mid-fetal stage, revealing that (i) OPCs were more similar than neurons when comparing the anatomical origin (cortex vs. pons), and (ii) four oligo cell clusters were characterized, represented by proliferating OPCs (PDGFRα^+^/CDK1^+^), OPCs that exited cell cycle (PDGFRα^+^/CDK1^–^), OPCs starting to express APOD (apolipoprotein D), and newly formed OLs (NFOLs) expressing MOG, ITPR2, and PDGFA. These cell clusters followed a stepwise developmental path with NFOLs enriched by the end of the second trimester. Similar to mice, OLIG1 was expressed throughout OL development, PDGFRα was specifically expressed in OPCs, and myelination proteins such as MBP appeared when OPCs differentiated into OLs (Fan et al., 2020). A further elucidation of the developmental landscape of human early OPC was recently shown, using engineered knock-in hESC-reporter lines, with a tag under the endogenous, OPC-specific, PDGFRα promoter. Time-course sc RNA-sequencing of purified OPCs uncovered transcriptional heterogeneity of PDGFRα^+^ OPCs; pseudotime analysis allowed to identify two distinct developmental trajectories for OLs vs. astrocytes differentiation from OPCs, and highlighted mTOR and cholesterol biosynthesis as key signaling pathways in the maturation of OLs from OPCs (Chamling et al., 2021). Moreover, in another study, authors applied sc RNA sequencing to OL-lineage cells isolated from surgical tissues over second-trimester fetal, 2-year-old pediatric, 13-year-old adolescent, and adult donors, thereby covering advanced developmental stages including time of peak myelination. Three distinct cellular subpopulations were identified, including an early OPC group, enriched for the premyelinating fetal group of cells, a late OPC group (l-OPC) and a mature OL group, two cell clusters present in both the pediatric and adult age group. However, single cell analysis revealed that while pediatric vs. adult l-OPCs differ significantly for their molecular features, mature OLs are very similar across age groups (Perlman et al., 2020).

## Models to Study Human Oligodendrocytes in Physiology and Disease

Before the advent of the hiPSCs technology 15 years ago establishing valid and reproducible methods to culture human cells *in vitro* presented major difficulties. In the case of the CNS, these difficulties were amplified by the anatomically difficult access to human brain tissue; studies were therefore almost exclusively limited to either postmortem or “normally appearing” cerebral samples from surgeries. Besides the problem related to the unpredictability of sampling timing, studies performed with such samples suffered from unsurmountable tissue degradation and low reproducibility of results, and their relevance was highly limited to specific pathophysiological conditions. Moreover, in these studies, it was difficult to provide the appropriate control tissue from healthy individuals, nor it was possible to follow the development of a disease in the same patient longitudinally or to predict how his/her brain would respond to therapies. The only alternatives were represented by immortalized human CNS cell lines, or by a very limited repertoire of human primary cell cultures. These two options also have significant issues; in the former, the *in vitro* “immortalization” procedures (that necessarily included the use of oncogenes) can induce unpredictable changes of cell behavior, whereas, in the latter, human primary cells invariably underwent aging when placed in culture and could rarely be kept in culture for more than a few passages. All these difficulties have severely hampered progress of knowledge in human OL biology.

### Deriving Human Oligodendrocytes From Pluripotent Stem Cells

A first progress was made with the discovery of ESCs, pluripotent cells directly extracted from embryos, which could be committed to neural progenitors and then differentiated *in vitro* into astrocytes, neurons or OLs. Despite human ESCs have been widely used to unravel fundamental questions of OL development and to shed light on the mechanisms driving CNS cell differentiation and behavior, their application has been profoundly limited by ethical concerns associated to their origin, their scarce availability, low reproducibility of the published protocols, also across different hESC lines, and from their low efficiency to generate mature myelinating OLs (Alsanie et al., 2013). In this context, the introduction of the iPSCs technology, namely the possibility of reprogramming somatic cells into pluripotent cells by transduction using a defined set of transcription factors (Teshigawara et al., 2017), and their subsequent *in vitro* differentiation to the desired cell phenotype, greatly improved the molecular and functional characterization of human OLs and, more in general, it opened the unexpected opportunity to study the unique behavior of any cell type in a personalized way, acquiring information on how diseases develop in every single individual, and allowing the identification of the very appropriate cure for that disease in that specific patient (Figure 1E).

**FIGURE 2:**
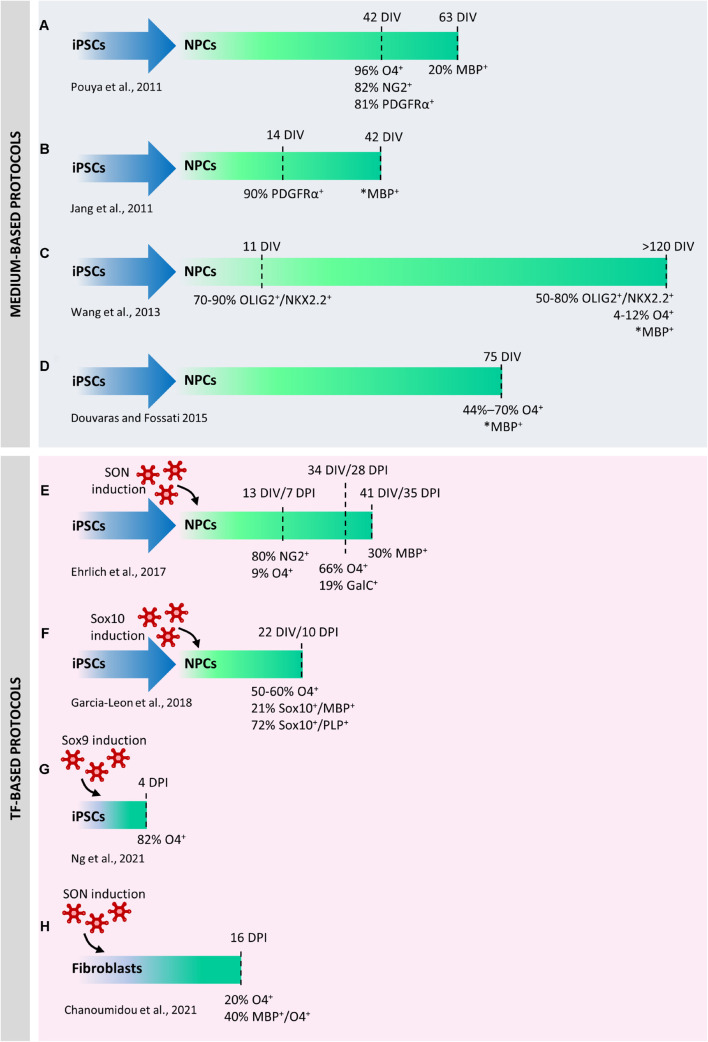
Comparison between protocols used to differentiate human induced pluripotent stem cells (iPSCs) into oligodendrocytes (iOLs). The first published protocols were exclusively based on chemically defined media supplemented with mitogens or differentiation promoting factors **(A–D)**. Medium-based protocols were progressively refined to increase their robustness and reproducibility in a higher number of lines. Direct differentiation by ectopic expression of selected transcription factors has significantly accelerated the differentiation of neural precursor cells (NPCs) into iOLs and increased the reprogramming efficiency **(E–G)**. In one case, direct conversion of iPSCs into iOLs has been described **(H)**. DIV, days *in vitro*; DPI, days post induction. *Percentage not available.

Since the discovery of iPSC, several groups have developed specific protocols to obtain hiPSC-derived OLs by adopting protocols previously used to derive hESC-derived OLs. These protocols were exclusively based on different chemically defined media supplemented with mitogens (e.g., bFGF, PDGF and EGF) or differentiation promoting factors (e.g., RA, T3) at proper time intervals in the attempts to recapitulate *in vitro* the developmental cues that occur *in vivo* during oligodendrogenesis (Jang et al., 2011; Pouya et al., 2011). Indeed, sequential induction steps allow to obtain embryoid bodies, to acquire a neural phenotype and finally to drive their differentiation through the oligodendroglial lineage. However, these medium-based protocols have a limited efficiency, require long culture periods (42 days were necessary to obtain immature O4^+^ cells from hiPSCs, and other 3–4 weeks to further differentiate them into MBP^+^ cells (Figures 2A,B) and have been tested on a reduced number of hiPSCs lines. Based on these earlier studies, more recent papers have developed more robust differentiation protocols (Figures 2C,D), in which, despite the still long differentiation times (70–100 DIV to generate functional OPCs/OLs from hiPSCs) the results were more consistent and were validated on an higher number of lines (Wang et al., 2013; Douvaras et al., 2014; Douvaras and Fossati, 2015).

Novel approaches based on the ectopic over-expression of transcription factors (TFs) completely changed the view of hiPSC reprogramming to OLs. A first protocol published in 2017 was based on the conversion of hiPSC-derived neural progenitor cells (hiNPCs) into hiOLs by transducing them with a “SON construct” consisting in the combination of three typical oligodendroglial TFs, namely SOX10, OLIG2, and NKX6.2 (Ehrlich et al., 2017). This approach yielded up to 70% O4^+^ OLs within 28 days of differentiation and 30% of the O4^+^ cells further differentiated into mature MBP^+^ OLs within seven additional days (Figure 2E). In a subsequent study (Garcia-Leon et al., 2018), the expression of SOX10 alone led to the production of approximately 50%–60% of O4^+^ cells and 21% of MBP^+^ mature hiOLs in 22 days from hiPSC stage (Figure 2F). Global gene-expression profiling and RNA-sequencing (RNA-seq) performed on SON- and SOX10-induced O4^+^ cells demonstrated that hiOLs generated by TF overexpression resemble primary human adult OLs at the transcriptome level. O4^+^ hiOLs were also able to myelinate axons both *in vivo* after transplantation into Shi/Shi Rag2^–/–^ dysmyelinating immunodeficient mice and *in vitro* when co-cultured with neurons derived from hiPSCs or nanofibers (Ehrlich et al., 2017; Garcia-Leon et al., 2018, 2020).

Very recently, a seminal paper identified 241 TFs whose ability to induce cell differentiation had never been previously investigated (Ng et al., 2021). Several of them were virtually able to downregulate the expression of pluripotency markers in a few days. Among them, authors demonstrated that ATOH1, NKX3-1, ETV2 and SOX9 were able to directly reprogram hiPSCs into neurons, fibroblasts, vascular endothelial-like cells and OLs, respectively, only in 4 days (Figure 2G). In addition to the incomparable speed of the protocol, full cell reprogramming can be achieved in serum-free media without any supplements, soluble factors, or mechanical cues, thus allowing the parallel programming of hiPSCs into two or three defined cell types in the same culture. Four days after Sox9 infection, 82% of the cells were O4^+^ and, similarly to what observed with Sox10- and SON-mediated induction, their transcriptomic signature was similar to primary OLs. Parallel induction of OLs and neurons in the same plate demonstrated that these co-cultures could form compact myelin after 30 days.

Finally, another outstanding paper (Chanoumidou et al., 2021) has described a robust and highly reproducible protocol for the direct conversion of human young, adult, and old-aged fibroblasts into OLs by overexpressing the SON construct for 16 days (Figure 2H). From 9 days post infection on, directly converted human induced OL-like cells (dc-hiOLs) progressively acquired a branched morphology upon medium enrichment with differentiation factors (including NT3, insulin-like growth factor 1, and dibutyryl cyclic). Flow-cytometry and immunocytochemistry analyses revealed that O4^+^ cells were already detectable 8 days post-infection and their number increased progressively until day 16 (as well MBP^+^ cells), reaching an average percentage of 20% between cells derived from young, adult and old-aged fibroblast. Similarly to hiOLs derived from the transduction of NPCs with SON (Ehrlich et al., 2017), dc-hiOLs were able to ensheath synthetic nanofibers as well as neuronal processes of hiPSCs derived neurons.

Apart from the different protocol length, the hiOLs generated in these studies showed similar properties and myelinating capability. The discrepancy in the time needed to achieve an efficient re-programming could be explained by the differences in the infected cells (NPCs vs. iPSCs) and the concomitant different number, type and role of TFs used in the different protocols (three vs. one TF and Sox10 vs. Sox9). Indeed, whereas Sox9 has a central role in the specification of OPCs and continues to be expressed until terminal differentiation, Sox10 appears immediately after specification, and plays an essential function in the induction and execution of the terminal differentiation and myelination program (Reiprich et al., 2017).

The use of TFs in cell reprogramming to generate human OLs is a relatively new approach, and it is likely that different combinations of TFs will enable to further improve the generation of hiOLs not only in terms of yield, but also to reproduce the functional heterogeneity of OLs and their precursors.

### Modeling Human Diseases Using Induced Pluripotent Stem Cells-Derived Oligodendrocytes

The studies described above represented an important proof of concept of the broad and paramount applications of these cells in pre-clinical research and paved the way for their applications in high-throughput screening for drug discovery (see section “Applications of Induced Pluripotent Stem Cells-Derived Cells and Cerebral Organoids to Drug Discovery: Advantages and Challenges”) and in modeling human demyelinating diseases, in particular those with a genetic component. This is the case of the Pelizaeus-Merzbacher disease (PMD), an X-linked leukodystrophy characterized by developmental delay, ataxia, spasticity, and nystagmus. Different signs of the disease are due to a constellation of different mutations in the Proteolipid Protein 1 (PLP1) gene that encodes for one of the major myelin proteins. Aberrant expression or misfolding of this protein leads to its accumulation in OLs and eventually to apoptosis and demyelination in many areas of the CNS (Nobuta et al., 2019).

Despite several efforts have been made to identify a common pathogenic process underlying this disease, no direct links between an individual’s unique PLP1 mutation and the etiology or course of their disease have been found.

Immunocytochemistry and flow cytometry analysis on hiOL cultures derived from PMD-hiPSCs (Douvaras et al., 2014; Douvaras and Fossati, 2015) allowed to get insights into cellular and molecular defects underlying the disease (Nevin et al., 2017). The hiOPC number was strikingly reduced in most cultures, suggesting that, at least in a subset of individuals, defects at the OPC stage anticipate dysmyelination. Moreover, despite OPC deficits, most PMD cultures (10 out of 12) were still able to produce O4^+^ OLs, but these immature cells were noticeably defective in process extension and branching. In the cultures in which PLP was detectable, the accumulation was always in the ER (Nevin et al., 2017). Different missense mutations showed accumulation and mis-localization of PLP to the ER, increased ER stress, apoptosis and decreased myelination, consistent with the different level of clinical severity of the subjects (Numasawa-Kuroiwa et al., 2014).

Mutant iOLs also showed lipid peroxidation, abnormal iron metabolism, and hypersensitivity to free iron (Nobuta et al., 2019) when compared to line-specific isogenic controls obtained by correcting the PLP1^G74E^ mutation in patient’s iPSCs, thus suggesting a causal link between ferroptosis and PMD pathogenesis. This hypothesis was further confirmed by the observation that treatment with deferoxamine (DFO), deferiprone (DFP), two Food and Drug Administration (FDA)-approved iron chelators, and apotransferrin (apo-TF) rescued mutant OL apoptosis, survival and differentiation *in vitro*. Human mutant O4^+^ cells pre-treated with apo-TF or DFO during *in vitro* differentiation and transplanted into the cerebellum of immunocompromised Shi mice or placed on human fetal brain slice cultures showed also a substantially improved differentiation (Nobuta et al., 2019). The efficacy of DFP in enhancing OL survival and promoting new myelin formation was finally demonstrated in Jimpy mice, a PMD mouse model that harbors a Plp1 point mutation and causes a very aggressive disease.

Thus, this article highlighted an additional cardinal feature of hiOLs, namely their translational importance in paving the way to the discovery of new effective drugs for still incurable CNS diseases.

The iPSC technology has been instrumental to study OL behavior also in non-genetic diseases, such as MS. Myelin-forming OLs can be derived *via* hiPSCs from both relapsing-remitting (RRMS) (Song et al., 2012) and primary progressive (PPMS) MS patients. However, their maturation and myelination capabilities do not seem to be significantly different from those of healthy OLs (Song et al., 2012; Douvaras et al., 2014; Garcia-Leon et al., 2018; Mozafari et al., 2020; Starost et al., 2020). Conversely, supernatants from both polarized human microglia or activated peripheral blood mononuclear cells (PBMCs) significantly inhibited hiOL differentiation, highlighting a role for immune regulators as extrinsic key players in MS pathogenesis and potentially responsible of repair failure (Hess et al., 2020; Starost et al., 2020). In addition, hiPSC-NPCs from patients with PPMS were less able to provide neuroprotection to myelin injury or support OPC differentiation *in vitro* compared to control iPSC-NPCs, probably due to their aberrant cellular senescence (Nicaise et al., 2017, 2019). Since cellular senescence can be associated with a proinflammatory and paracrine effect exerted by the cells on surrounding tissue, this can be a further extrinsic mechanism contributing to the not permissive environment within white matter lesions, leading to remyelination impairment.

Thanks to hiPSCs, it has been possible to highlight OL involvement in diseases such as amyotrophic lateral sclerosis (ALS), schizophrenia, and frontotemporal dementia with Parkinsonism, in which demyelination is not traditionally a key feature (Chanoumidou et al., 2020).

Amyotrophic lateral sclerosis is characterized by the progressive loss of motor neurons (MNs) in the brain cortex and spinal cord, leading to muscle atrophy and death by respiratory paralysis within 3–5 years after the first symptoms (Brown and Al-Chalabi, 2017). Recent studies on mouse models of ALS have shown that OLs are also involved in the non-cell-autonomous nature of the disease contributing to disease progression by triggering or aggravating damage to MNs (Kang et al., 2013; Philips et al., 2013) [for extensive review, see Raffaele et al. (2021a)]. Analysis on hiOLs derived from human sporadic and familial ALS patients carrying mutations in SOD1, TARDBP, C9orf72, and FIG4 allowed to demonstrate that, while these cells were able to differentiate and maturate similarly to matched controls, they exerted toxic effects on mouse motor neurons *in vitro*, partially due to defective lactate production and release by hiOLs. However, addition of lactate to cocultures only partially reversed the ALS-hiOLs mediated toxicity, suggesting that MN survival also requires additional insoluble factors and/or cell-to-cell contacts (Ferraiuolo et al., 2016).

## Brain Organoids as New Experimental Systems for *In Vitro* Myelination Studies

The interest for recapitulating *in vitro* the complexity of human brain development ushered the development of organoids, 3D structures grown from stem cells and consisting of organ-specific cell types that self-organizes and can be maintained in culture over extended periods of time (Di Lullo and Kriegstein, 2017; Lopez-Tobon et al., 2020). Brain organoids can be derived from both pluripotent stem cells, ESCs and iPSCs. Starting from the pluripotent state, most of the organoid protocols are characterized by the generation of embryoid bodies (EBs) as a first step: monolayers of pluripotent stem cells growing on a matrix are dissociated at single cell level, and a precise number is replated into ultra-low attachment wells. This enables the cells to aggregate and form EBs that grow in suspension and are subsequently patterned toward the lineages of differentiation of interest. There are several approaches that aim at recapitulating different regions and/or complexity of the CNS development, each of which is better suited for addressing specific biological questions. However, there are common aspects to all brain organoid protocols, which include (i) the formation of self-organizing structures, in particular rosettes which resemble the embryonic stratification of the epithelium of the neural tube with apico-basal polarity and are subdivided into proliferative and differentiating zones, (ii) the generation of different subpopulations of progenitors usually absent in the 2D counterparts and (iii) a considerable degree of basic compartmentalization at the extracellular level that includes the production of their own rudimentary ECM (Kelava and Lancaster, 2016; Quadrato et al., 2016; Di Lullo and Kriegstein, 2017; Giandomenico and Lancaster, 2017; Kyrousi and Cappello, 2020).

Different brain organoids can be on the other hand very different in their specificity to recapitulate either whole brain or specific brain regions such as cortex, hippocampus, thalamus or hypothalamus (Qian et al., 2016; Birey et al., 2017; Lancaster et al., 2017; Xiang et al., 2019; Ozaki et al., 2021). In case of cerebral cortex, it is possible to differentiate the most dorsal part of the forebrain (the pallium), that gives rise to glutamatergic neurons, or the ventral one (the subpallium) that gives origin to GABAergic interneurons (Bagley et al., 2017; Birey et al., 2017). In this way, 3D differentiation of hiPSCs into cortical brain organoids can recapitulate the physiological emergence of the six distinctive neuronal layers of the cortex, along with astrocytes (Sloan et al., 2017), and the outer radial glia subset of progenitors that underlies the specific expansion of the human neocortex (Bershteyn et al., 2017; Pollen et al., 2019). Brain organoids reproduce the cell diversity of human brain, they show patterns of electrical activities whose oscillations resemble the prenatal human electroencephalography (Trujillo et al., 2019), and, when transplanted in mouse, they establish functional connections with different regions of the host brain (Mansour et al., 2018) and innervate dissected mouse spinal cord inducing muscle contraction (Giandomenico et al., 2019).

**FIGURE 3:**
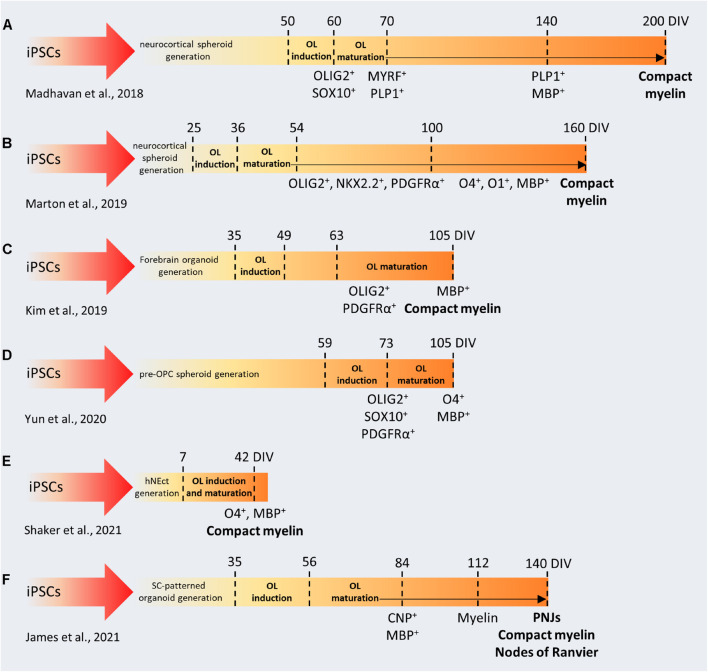
Comparison between protocols used to generate brain organoids containing myelinating oligodendrocytes. **(A–F)** In the first phase of each protocol, iPSCs are cultured and patterned to generate 3D brain-like structures (forebrain, spinal cord, neuroectoderm). Then, OL induction and maturation is achieved by using specific soluble factors (e.g., PDFG-AA, IGF1, FGF2, T3). iPSCs, induced pluripotent stem cells; DIV, days *in vitro*; OL, oligodendrocyte; OPC, oligodendrocyte precursor cell; SC, spinal cord; hNEct, human neuroectodermal layer; PNJs, paranodal axoglial junctions.

Brain organoids have been used to study the molecular basis of human corticogenesis (Lopez-Tobon et al., 2019), to develop new disease models (Birey et al., 2017; Caporale and Testa, 2019; Klaus et al., 2019; Cheroni et al., 2020; Khan et al., 2020), and to study evolution (Kanton et al., 2019; Pollen et al., 2019) usually focusing on neurons and on astrocytes. The OL lineage remained underrepresented because, up to 2018, no protocol has yielded reproducible generation and maturation of OLs. Despite the neural origin of OPCs (Naruse et al., 2017), organoids that are patterned toward the dorsal pallium do not generate OPCs in a robust way, and, consequently, they have never been used to study myelination. This paradigm changed after the optimization of protocols in which cortical brain organoids were patterned toward the dorsal telencephalon (Pasca et al., 2015), through timely exposure to platelet-derived growth factor AA (PDGF-AA) and insulin-like growth factor 1 (IGF-1) to drive the expansion of native OPC populations, followed by thyroid hormone (T3) to induce OL differentiation (Madhavan et al., 2018; Marton et al., 2019). All these protocols (Figures 3A,B) were validated across different hiPSC lines and characterized at the level of single cell omics to identify the stages and trajectories of OL differentiation, showing similar expression patterns compared to human primary cells.

In the rodent brain, the OL first wave of differentiation derives from the ventral regions, but dorsal-derived OLs are the ones required for proper myelination in the brain cortex. The analogous developmental processes and timing in humans was unknown until recently, when Kim et al. (2019) took advantage of OLIG2-GFP knock-in hiPSC reporter lines to follow oligodendrogenesis in forebrain organoids patterned toward the ventral and the dorsal pallium (Figure 3C). With this approach, they identified distinct temporal expression patterns in the two lineages, even though OLs could be differentiated in both systems, after neuronal maturation. When assembling ventral and dorsal organoids, dorsally derived oligodendroglial cells outcompete ventrally derived oligodendroglia and become dominant after long-term culture (Kim et al., 2019). These results are in line with recent observations about the specific impact of ventral OPCs on the migration and maturation of mouse cortical interneurons (Lepiemme et al., 2021). These pioneering studies demonstrated that generating brain organoids with myelinating OLs is indeed possible, however, they require very long protocols (from 110 to 210 days) to produce myelinated fibers. Interestingly, it has been shown that pre-patterning of NSCs with FGF2, PDGF-AA, and purmorphamine, leads to the production of OLIG2 and NKX2.2-expressing neurospheres (Figure 3D) and facilitates glial specification during organoid development (Yun et al., 2020). These OPC-derived spheroids were used to validate the effect of benztropine and miconazole, two promyelinating drugs *in vivo* (Deshmukh et al., 2013). Both drugs dramatically enhanced the MBP^+^/SOX10^+^ populations compared to DMSO-treated organoids after 12 weeks in culture, while miconazole also showed a 15% increase in terms of myelinating OLs compared to T3-treated organoids. Recently, Shaker et al. (2021) developed a protocol to generate brain organoids containing OLs capable of myelinate endogenously produced cortical neurons in only 42 days (Figure 3E), through the exposure to a cocktail of growth factors and small molecules. This contains T3, neurotrophin NT3, hepatocytes growth factor, IGF-1, and PDGF-AA to pattern the differentiation toward the oligodendroglial lineage and to foster the proliferation of OPCs, B27 without vitamin A and cAMP to drive the maturation of OPCs to myelinating OL, biotin and BME to increase the survival of the maturing OLs, as well as N2, insulin, and non-essential amino acids to promote the co-differentiation of neurons and astrocytes (Shaker et al., 2021).

As mentioned above, regional identity of developing OLs is another important factor that may influence the timing and the myelinating properties of OLs during organoid formation. During development, MBP^+^ forebrain-derived OLs emerge at gestational week 17–20, but myelination starts several months later, in the postnatal phase (Jakovcevski et al., 2009). In contrast, the emergence of ventral spinal cord-derived OLs occurs much earlier, around gestational week 10, and is followed by the appearance of myelinated fibers 4 weeks later (Weidenheim et al., 1993). Similarly, organoids generated from the ventral spinal cord showed earlier myelination (Figure 3F). Moreover, electron microscopy revealed that in these organoids, called “myelinoids,” myelin thickness, compaction and assembly at the paranodal junction are comparable to those observed *in vivo* (James et al., 2021), whereas forebrain-patterned organoids demonstrated sparse myelination and lacked the typical architecture of myelinated axons. These parameters are crucial to model myelin physiology and to recapitulate myelin function. Myelinoids were also used to investigate the concept of adaptive myelination, according to which OLs modulate myelin structure in response to changes in neuronal activity. Myelinoids chronically treated with tetanus toxin, which blocks synaptic vesicles release, showed a 20% reduction in the number of sheaths made by individual OLs and a 38% reduction in myelin volume. Due to their spinal origin, myelinoids may not be optimal to recapitulate myelination kinetics or neurological conditions typical of the brain, but, indeed, they represent an important proof-of-concept in the field.

As shown for iPSCs, human brain organoids can be exploited to study cellular pathology and dysfunction associated to genetic mutations, with the advantage of having a complex architecture. Oligocortical spheroids generated from iPSC lines with different PMD-related mutations (PLP1 deletion, duplication and point-mutation) have shown PLP1 levels and PLP1 localization correlated with the different genetic status of the donors and alteration in the number of differentiating OLs (Madhavan et al., 2018). Interestingly, CRISPR correction of the PLP1 point mutation in iPSCs prior to spheroid generation restored PLP1 mobilization and OL number. OLs in brain organoids are also susceptible to lysolecithin (Marton et al., 2019), suggesting that they can be used to study OL loss or injury in disorders affecting CNS myelination.

The examples described above indicate that, although the application of brain organoids to myelination studies is still in its infancy, further optimization of this technology could allow the development of totally new experimental platforms to better understand human myelin biology, to investigate the basis of adaptive myelination, to generate new *in vitro* demyelination/remyelination models, to study patient-specific genetic background as an intrinsic trigger of myelin dysfunction and to perform drug screening (Figure 1F).

## Applications of Induced Pluripotent Stem Cells-Derived Cells and Cerebral Organoids to Drug Discovery: Advantages and Challenges

Due to their capability to recapitulate several pathophysiological aspects of the bona fide human cells, one of the most obvious applications of hiOLs has been their use as a tool for phenotypic drug screening, thus allowing to validate data previously obtained on either rodent OLs or more limited human OL models. For example, Ehrlich et al. (2017) cultured hiOL in the presence of miconazole, clobetasol, benztropine, indometacin, clemastine, and oxybutynin, six FDA-approved drugs used for the treatment of various diseases and acting with different mechanisms of actions, that have been recently described to promote differentiation or myelination of rodent OLs, thus highlighting their potential in drug repositioning (Deshmukh et al., 2013; Mei et al., 2014; Najm et al., 2015). However, only miconazole, clobetasol and benztropine did increase the percentage of MBP^+^ mature hiOLs after 21 days in culture, suggesting that species-specific differences could be relevant for drug screening (Ehrlich et al., 2017). Accordingly, benztropine and clemastine enhanced the terminal differentiation and maturation also of hiOLs derived from direct conversion of fibroblast with SON construct, while miconazole showed a milder but still significant effect (Chanoumidou et al., 2021). High-throughput screening (HTS) applied to human iPSC-derived neurons and OLs co-culture also demonstrated enhanced myelination after treatment with miconazole, clobetasol and pranlukast (Garcia-Leon et al., 2018). Interestingly, drugs previously shown to promote oligodendroglial differentiation failed to restore impaired hiOL differentiation induced by PBMC supernatants, whereas immunomodulatory treatments on PBMCs partly restored hiOL differentiation, indicating that more complex models are needed to mimic the inflammatory environment typical of MS lesions (Starost et al., 2020).

Thus, one first conclusion that can be drawn from these data is that drug screening on human iOL (and more in general on human iPSC-derived cells) can help surmounting species related differences in pharmacological responses, which is not a trivial issue.

Second, generating patient-specific terminally differentiated cell types offers the possibility to conduct *in vitro* “clinical trials” (McNeish et al., 2015), in which drugs can be tested for both efficacy and toxicity. This can guide the choice of the most appropriate treatment for each patient and overcome problems related to individual susceptibility to develop side effects, although, of course, caution should be placed on the issue that differentiated iPSC-derived cell systems cannot reveal propensity to side effects related to individual drug metabolism and excretion. Moreover, *in vitro* clinical trials can lead to improved patients’ stratification and lower compound attrition rates. Certainly, the availability of a model allowing “personalized” drug screening will greatly facilitate the drug discovery process that normally takes more than 10 years to go from the discovery of a new molecule to its approval.

Third, besides the development and screening of new drugs, iPSC-based models can be used to identify already marketed drugs (or drugs for which safety studies on humans are already available) to be repurposed for the treatment of orphan diseases with high medical need. As an example, ezogabine, an anti-epileptic drug, showed efficacy in iPSC-derived motor neurons to model ALS, leading to the subsequent initiation of clinical trials (McNeish et al., 2015).

However, there are still issues to be solved. The first is related to the molecular maturity of iPSC-derived cells, which exhibit immature characteristics comparable to embryonal or fetal cells. This is particularly important for modeling late-onset diseases, where maturity is a critical aspect, like Alzheimer’s and Parkinson’s disease. In order to simulate aging *in vitro*, scientists are tackling this problem using chemical and pharmacological approaches reproducing some of the features typical of aged tissues, for example by employing specific culture medium formulations inducing mitochondrial stress, inhibiting protein catabolism, or overexpressing progerin, a truncated version of the premature aging protein lamin A (Doss and Sachinidis, 2019).

A second issue is that, inevitably, drug screening performed on iPSC-derived OLs does not consider the contribution of other cell types to drug response (see also above) raising the need of setting up more complex models, where all the cell types of the living brain are present and interact with each other.

In this respect, cerebral organoids (COs) show substantial potential, and, as underlined above, accumulating data are validating organoids as a reliable model. Recently, a human CO model of prion disease was used for evaluating the effectiveness of putative drugs in inhibiting prion accumulation (Groveman et al., 2021). Another recent study generated 1,300 iPSC-derived COs from 11 sporadic AD patients to identify FDA-approved drugs as candidates for drug repositioning (Park et al., 2021). Cocultures of motor neuron spheroids from a patient with sporadic ALS and 3D muscle fiber bundles were used to reproduce ALS pathology and to test the efficacy of candidate drugs already used in phase 1 and 2 clinical trials (Osaki et al., 2018).

However, there still remain several challenges that are currently being addressed by academic and industrial researchers in order to fully exploit this technology to the discovery of new CNS drugs, including new myelinating therapeutic agents (Salick et al., 2021). Here, we summarize some of them.

A first issue relates to the need of generating *automated systems* to overcome the very high experimental variability between different cerebral organoids preparations. There is a need to develop fully integrated end-to-end cell culture systems (incubator and imaging platforms) that can maintain, passage and differentiate cells to generate uniform cerebral organoids in an automated manner to reduce the variability introduced by multiple human hands. A second issue relates to the need of *high resolution and potent imaging systems*, which, differently from monolayered cells, are essential in the case of organoids, both to check their expected 3D conformation and to assess the effects of drugs and other treatments on organoids’ 3D organization. Traditional fluorescence microscopes are not suited for performing high resolution imaging of 3D samples deeper than few micrometers, mainly due to the obscuring effects of light scatter. This limitation can be overcome by different strategies: (i) clearing techniques have been developed to ensure a uniform density of the sample (through a combination of delipidation, decolourization, decalcification, and refractive index matching) so that all wavelengths of light can pass through the tissue (Richardson and Lichtman, 2015; Ueda et al., 2020). These approaches are often combined with light sheet microscopes for rapid, high-resolution imaging of large volumes, indeed they have been successfully applied to organoids, achieving imaging of large and thick samples with subcellular resolution (Boutin and Hoffman-Kim, 2015; Dekkers et al., 2019; Albanese et al., 2020). However, they are not suited for live imaging applications that are essential to study key neurodevelopmental processes; (ii) computational methods such as 3D deconvolution can assist 3D resolution, but there are still difficulties in deconvolving complex samples because widefield microscopy deconvolution provides limited penetration due to high background and light scattering (McNally et al., 1999); (iii) spinning disk confocal microscopy and resonant scanning microscopy have significantly improved point scanning microscopy, but while such systems enable to observe thick living samples at increased depth and speed, they all result in substantial phototoxicity, due to out-of-focus illumination (Jemielita et al., 2013); (iv) multiphoton excitation allows to acquire deep planes from thick samples with reduced phototoxicity because there is no absorption above and below the plane of focus and indeed is considered the best solution for imaging deep layers in the organoids when clearing cannot be performed (Dekkers et al., 2019). Deleterious effects are occasionally observed with two-photon excitation of certain fluorophores and imaging is relatively time consuming and may lead to photobleaching since it is still based on a point scanning approach.

Thus, proper microscopy systems maintaining high resolution, low phototoxicity and photobleaching, while increasing speed, depth, and size of the field of view, still need to be developed.

Finally, the *scale of experiments* required for high-throughput drug screening is an additional challenge for the cerebral organoid field. Biotech and pharma industry are just starting to explore this new area (Shah et al., 2020), but work is still ongoing to uncover the full potential of cerebral organoids in target and drug discovery.

## Conclusion

Myelination is a crucial process in vertebrates and its complexity has made the elucidation of the underlying mechanisms extremely challenging. The discoveries in the physiology of myelination have been possible only thanks to the advances in the *in vitro* technologies. The new frontiers are now humanized models, increasingly reproducing human physiology and pathology. However, human iPSCs are only a starting point. Indeed, hiOLs are sensitive to the environment, and their behavior can be influenced by the cells and cell structures they take contact with, including glia, vessels, axons, ECM, by cell culture media and grow factors, and by the mechanical properties of the culture supports. Brain organoids recapitulate the developmental trajectories of neural cells, circuits, and connection and are likely to become a new powerful tool to study myelination, demyelination and remyelination dynamics in the next few years. Myelinating/demyelinating models based on human iPSC-derived cells should be consolidated to become a new gold standard and progressively reduce the use of animal models. The development of new technologies for data analysis, automation, miniaturization and HTS will pave the way for new reliable drug screening platforms.

## Author Contributions

DM and MB prepared the figures. DL combined the contributions and revised the manuscript. All authors contributed to conception and writing of the present review and read, revised, and approved the submitted version.

## Conflict of Interest

The authors declare that the research was conducted in the absence of any commercial or financial relationships that could be construed as a potential conflict of interest.

## Publisher’s Note

All claims expressed in this article are solely those of the authors and do not necessarily represent those of their affiliated organizations, or those of the publisher, the editors and the reviewers. Any product that may be evaluated in this article, or claim that may be made by its manufacturer, is not guaranteed or endorsed by the publisher.
